# Transformation and Detoxification of Typical Metallurgical Hazardous Waste into a Resource: A Review of the Development of Harmless Treatment and Utilization in China

**DOI:** 10.3390/ma17040931

**Published:** 2024-02-17

**Authors:** Yuanhang Wang, Haiquan Zhao, Xinyu Wang, Junkai Chong, Xiangtao Huo, Min Guo, Mei Zhang

**Affiliations:** 1 State Key Laboratory of Advanced Metallurgy, School of Metallurgical and Ecological Engineering, University of Science and Technology Beijing, Beijing 100083, China; 2 School of Vanadium and Titanium, Panzhihua University, Panzhihua 617000, China

**Keywords:** metallurgical hazardous waste, stainless steel dust/sludge, aluminum ash, harmless treatment, resource utilization

## Abstract

The production process of the metallurgical industry generates a significant quantity of hazardous waste. At present, the common disposal method for metallurgical hazardous waste is landfilling, which synchronously leads to the leaching of toxic elements and the loss of valuable metals. This paper presents a comprehensive review of the research progress in the harmless treatment and resource utilization of stainless steel dust/sludge (including stainless steel dust and stainless steel pickling sludge) and aluminum ash (including primary aluminum ash and secondary aluminum dross), which serve as representative hazardous wastes in ferrous metallurgy and nonferrous metallurgy, respectively. Additionally, the general steps involved in the comprehensive utilization of metallurgical hazardous waste are summarized. Finally, this paper provides a prospective analysis on the future development and research trends of comprehensive utilization for metallurgical hazardous waste, aiming to offer a basis for the future harmless, high-value, resource-based treatment of metallurgical hazardous waste and the realization of industrial applications in China.

## 1. Introduction

A significant amount of solid waste is generated in the metallurgical industry and its associated sectors [1,2]. Among this, some metallurgical solid wastes, such as stainless steel dust/sludge (including stainless steel dust and stainless steel pickling sludge) and aluminum ash (including primary aluminum ash and secondary aluminum dross), are categorized as hazardous wastes due to their excessive content of toxic heavy metal elements, like chromium, lead and cadmium [3]. The current disposal method for metallurgical hazardous wastes is mainly concentrated in landfilling, which not only occupies a large amount of land resources, but also pollutes the groundwater and surrounding environment. In addition, these treatment methods also have a great impact on animals, plants, and human health due to the leaching of heavy metal elements during stacking [4]. Therefore, it is an urgent imperative to find effective solutions of harmless treatment and resource utilization for metallurgical hazardous wastes to achieve sustainable development aligned with green principles.

The metallurgical industry can be classified into two main branches, namely ferrous metallurgy and nonferrous metallurgy. Compared to conventional steel, special steel exhibits enhanced strength, toughness, physical properties, chemical properties, and biocompatibility. Clearly, special steel will be the development trend of ferrous metallurgy. Stainless steel is a representative type of special steel, which is widely used in chemical industry, paper making, medical equipment and other fields [5]. The main solid waste generated during the process of stainless steel smelting is stainless steel dust/sludge, which has been classified as hazardous waste due to its excessive chromium content. With the rapid development of the stainless steel smelting industry, the production of stainless steel dust/sludge is also increasing year by year. Aluminum is the most widely used metal material with the highest yield in the field of nonferrous metallurgy. Aluminum ash is the main industrial solid waste produced in the process of electrolytic alumina, metal aluminum processing, and waste aluminum recycling. Due to the excessive content of inorganic fluorides, aluminum ash has been officially classified as hazardous waste. According to the statistics, approximately 30 to 110 kg of aluminum ash is generated for every ton of aluminum production [6]. In 2022, the primary aluminum production of China reached 4.02 × 10^7^ t, and the estimated discharge of aluminum ash reached around 4 × 10^6^ t. Additionally, in 2021, China enacted the new hazardous wastes law that stipulates that the storage time for hazardous wastes cannot exceed one year within factories, which further increased the burden of metallurgical enterprises. Therefore, the harmless treatment and resource utilization of stainless steel dust/sludge and aluminum ash are urgent topics to address.

The stainless steel dust/sludge and aluminum ash contain toxic elements, like Cr, Ni and Pb [7,8,9]. These harmful elements pose a leaching risk while stacking or landfilling, potentially leading to soil and groundwater contamination [10,11]. In view of the potential harm, the stainless steel dust/sludge and aluminum ash have been clearly classified as hazardous wastes. On the other hand, heavy metal elements, for instance Cr and Ni contained in stainless steel dust/sludge and aluminum ash, are also regarded as important secondary resources. Therefore, the comprehensive utilization of stainless steel dust/sludge and aluminum ash can not only mitigate environmental pollution, but also realize the recovery of valuable metal resources. 

Hence, this paper selects stainless steel dust/sludge and aluminum ash as the representative hazardous waste in ferrous metallurgy and nonferrous metallurgy, respectively, providing a comprehensive overview of the current research and development trend in the field of harmless treatment and resource utilization. The forthcoming sections are structured to unfold in a logical sequence, commencing with the formation and detailed examination of the physicochemical characteristics of these wastes, followed by an assessment of their leaching toxicities. We then delve into the various treatment methodologies, contrasting the merits and demerits of hydrometallurgical and pyrometallurgical approaches. Ultimately, the general steps for achieving harmless treatment and resource utilization of metallurgical hazardous wastes are outlined, aiming to offer deeper insights into the utilization of metallurgical hazardous wastes and to promote the sustainable development of the metallurgical industry. This review was guided by the relevance to the treatment and utilization of hazardous wastes, the innovation in methodology, and the potential for practical application.

## 2. Toxicity Detection Methods and Identification Standards of Hazardous Waste

### 2.1. Toxicity Detection Methods

There are five main methods for toxicity detection of hazardous waste. Among them, the solid waste-extraction procedure for leaching toxicity-sulphuric acid & nitric acid method (HJ/T 299-2007) [12], solid waste-extraction procedure for leaching toxicity-acetic acid buffer solution method (HJ/T 300-2007) [13] and solid waste-extraction procedure for leaching toxicity-horizontal vibration method (HJ 557-2010) [14] were promulgated in China. Additionally, the Characterization of waste. Leaching. Compliance test for leaching of granular waste materials and sludges (EN 12457-3) [15] and the Toxicity Characteristic Leaching Procedure (TCLP) [16] are widely adopted abroad. Herein, they are characterized in Table 1.

### 2.2. The Identification Standards of Hazardous Waste

The identification standards for hazardous wastes—identification for extraction toxicity (GB 5058.3-2007) [17], the Resource Conservation and Recovery Act 40 CFR 261.24 [18], and the European Environment Agency Directive 91/689/EEC [19] are the identification standards for hazardous waste in China, the US, and the European Union (EU), respectively.

#### 2.2.1. Identification Standards for Hazardous Wastes—Identification for Extraction Toxicity (GB 5058.3-2007)

Table 2 is the identification standards for hazardous wastes—identification for extraction toxicity (GB 5058.3-2007). According to the solid waste extraction procedure for leaching toxicity—sulphuric acid & nitric acid method (HJ/T 299-2007), if the concentration of any hazardous element in the leaching solution from the solid waste exceeds the concentration limit listed in Table 2, it is determined as hazardous waste with leaching toxicity characteristics.

#### 2.2.2. Resource Conservation and Recovery Act 40 CFR 261.24

Some metal limit values of 40 CFR 261.24 are shown in Table 3. Based on the Toxicity Characteristic Leaching Procedure (TCLP), if the concentration of any hazardous element in the leaching solution from the solid waste exceeds the concentration limit listed in Table 3, it is classified as hazardous waste exhibiting leaching toxicity characteristics.

#### 2.2.3. European Environment Agency Directive 91/689/EEC

Table 4 presents partial metal limit values for the European Environment Agency Directive 91/689/EEC. As shown in Table 4, solid waste is categorized into three types based on the concentration limit values, namely inert waste, non-hazardous waste, and hazardous waste.

## 3. State of the Art—Typical Ferrous Metallurgical Hazardous Waste

Stainless steel dust/sludge includes stainless steel dust and stainless steel pickling sludge. Stainless steel dust (SSD) and stainless steel pickling sludge (SSPS) are solid wastes produced in the stainless steel production process. The SSD and SSPS contain valuable metals, such as Fe, Cr, and Ni, which may pose leaching risks when they are stacked or landfilled. On the other hand, the Fe, Cr, and Ni contained in SSD and SSPS are also important secondary resources. Consequently, the harmless treatment and resource utilization of SSD and SSPS are particularly of concern.

### 3.1. Formation of SSD and SSPS

SSD is a mixture collected from various dust collection equipment during the stainless steel smelting process. It is mainly derived from the splashing of metal and slag in an electric arc furnace and argon oxygen furnace, and the volatilization of different elements at high temperature. Hence, SSD normally contains two species, namely electric arc furnace dust (EAF-D) and argon oxygen furnace dust (AOD-D).

SSPS is a kind of solid waste produced in the pickling process of stainless steel. The oxide layer formed during manufacture and processing reduces the corrosion resistance of stainless steel and adversely affects product performance [20]. In stainless steel production, a pickling passivation process is required to improve its surface quality [21]. A modern pickling process of stainless steel usually consists of a preliminary electrolytic pickling step in a neutral solution based on sodium sulfate (Na_2_SO_4_), followed by a chemical pickling step in a mixed solution of nitric acid (HNO_3_) and hydrofluoric acid (HF). The process concludes with thorough high-pressure water rinsing. The pickling wastewater produced during the rinsing of the steel product is usually treated with a reducing agent (e.g., NaHSO_3_) and lime milk, which reduces Cr^6+^ to Cr^3+^ and forms a precipitate (part of the sludge) with a variety of metal ions and F^−^ in the reaction tank, followed by solid–liquid separation in the filtrate reaction tank through flocculants. The wastewater is discharged after conditioning, and then the sludge is returned to the reaction tank, where it is concentrated and dewatered through a filter press to form SSPS [22].

### 3.2. Chemical Composition and Hazards of SSD and SSPS

#### 3.2.1. Chemical Composition of SSD and SSPS

The chemical composition of SSD from two enterprises is shown in Table 5. It can be seen from the table that valuable metals, such as chromium, nickel, and iron, are contained in SSD. The highest mass fractions of Fe_2_O_3_, Cr_2_O_3_ and NiO reach 51.44%, 15.19%, and 3.48%, respectively. The aforementioned observation demonstrates the recycling value of SSD.

The chemical composition of SSPS is shown in Table 6. In Table 6, the overall view is that the contents of CaO, Fe_2_O_3_, and CaF_2_ are high, and the metal oxides Fe_2_O_3_, Cr_2_O_3_, and NiO are relatively high, which means that they are rich in recovery value [28,29,30,31,32]. In addition, the chemical composition of SSPS generated by different steel mills and processes varies greatly; for example, some steel mills have a high SiO_2_ content in stainless steel pickling sludge, which also results in a high content in the subsequent recycling [33]. It is worth mentioning that SSPS contains a large amount of CaF_2_; this can be attributed to the use of hydrofluoric acid (HF) in the pickling process. HF is commonly used to remove oxide scale and other impurities from stainless steel surfaces. During the pickling process, HF reacts with the oxides on the steel surface, forming calcium fluoride (CaF_2_) as a byproduct.

#### 3.2.2. Hazards of SSD and SSPS

According to the National Hazardous Waste List (2021 edition), SSD and SSPS are classified as hazardous wastes (China 2021). Typically, about 18–33 kg of SSD [34] and 30–50 kg of SSPS [35] are generated per ton of stainless steel production, which contains Ni, Cr, CaF_2_, and other substances that pose a great potential threat to human health and the ecological environment.

Nickel contamination in the environment can damage ecosystems and directly cause crop yield reductions [36]. The high concentrations of nickel can irritate the skin and cause damage to lung, liver, and heart function [37]. The chromium contamination occurs in soil, water, air, and food, eventually entering the skin, bronchial tubes, and intestines [38]. The Cr^6+^ exhibits genotoxic and carcinogenic properties, causing damage to the human skin, respiratory tract, liver, and kidneys. On the other hand, a deficiency of Cr^3+^ is associated with various health issues, including elevated blood sugar, increased cholesterol levels, as well as diseases, such as heart disease and obesity [39]. The solubility of CaF_2_ in water is limited, with low toxicity, and it is easily absorbed by plants. However, the landfill disposal of SSD and SSPS may result in the release of elemental fluoride into water bodies and soil, posing potential threats to both the ecological environment and human health [40]. In general, harmless treatment should be carried out for SSD and SSPS before stacking or landfill.

### 3.3. Toxicity Leaching of SSD and SSPS

#### 3.3.1. Toxicity Extraction Test of SSD

Wang et al. [26] used the solid waste extraction procedure for leaching toxicity—sulphuric acid & nitric acid method (HJ/T 299-2007) to test the leaching capacity of harmful elements in SSD. The test results are presented in Table 7. It is evident that the concentrations of total Cr and Cr^6+^ are 19.40 mg/L and 18.60 mg/L, respectively, which exceed limit values of the identification standards for hazardous wastes—identification for extraction toxicity (GB 5058.3-2007). Consequently, SSD is classified as a hazardous waste.

#### 3.3.2. Toxicity Extraction Test of SSPS

Su et al. [21] conducted a toxicity leaching test of the harmful elements in SSPS by using the solid waste extraction procedure for leaching toxicity—sulphuric acid & nitric acid method (HJ/T 299-2007). The test results are shown in Table 8. It can be seen from the table that the leaching amount of chromium in SSPS was 15.2 mg/L, which exceeded the concentration limit of 15 mg/L in the identification standards for hazardous wastes—identification for extraction toxicity (GB 5058.3-2007). Therefore, SSPS was categorized as a hazardous waste.

### 3.4. Harmless Treatment of SSD and SSPS

At present, the comprehensive utilization of SSD and SSPS mainly includes harmless treatment and resource utilization. The harmless treatment of SSD and SSPS mainly involves the following two processes.

#### 3.4.1. Solidification Process

The solidification of SSD and SSPS involves uniformly mixing SSD and SSPS with solidification agents, and then the mixture is treated under a high temperature. The SSD and SSPS can be landfilled after solidification treatment. The commonly used solidification agents are cement, asphalt, lime, glass, and plastic. The cost of the landfill after solidification method for treating SSD and SSPS is relatively low. Tests have confirmed that the heavy metal ions after high-temperature treatment can be wrapped up by the solidification agent and become relatively stable. This method is simple and makes it easy to realize the harmless treatment of SSD and SSPS, and the treated SSD and SSPS meet the landfill standard of the environmental protection department [41]. Singhal et al. [42] made a cement–sludge cube with SSPS and cement. The compressive strength of this cube decreased with the increase in SSPS content. At the same time, after the TCLP test, there was almost no leaching of heavy metal chromium and nickel after 28 days of solidification of the cement–sludge cube. Tang et al. [43] mixed SSD and clay additive at a mass ratio of 1:1, and then the mixture was heat-treated at 1100 °C for 15 min to obtain a stable silicon–aluminum-based clay, and its leaching rate was lower than the environmental emission standard.

#### 3.4.2. Vitrification Process

The continuous Si-O bonds in the dense network structure of the vitreous body can fix a large amount of heavy metal elements. Studies have shown that chromium is stably distributed in the glass network in the form of complex anions [44]. Pelino et al. [45] mixed the electric arc furnace dust (EAF-D) with glass fragments and sand to prepare glass with different compositions, and then the stability of the prepared glasses under different EAF-D content was investigated. When the EAF-D content was 45%, the chemical stability and the chromium fixation effect of the prepared glass were the best. Ma et al. [46] used SSD as the research object, and different melting temperatures as well as holding times were set to prepare glassy products. The results showed that a vitrified product characterized with dense and homogenous structure, low porosity, and a smooth appearance could be made by means of an appropriate technique (temperature of 1450 °C; holding time of 0.5 h; alkalinity of 0.14–0.55). The toxic substances in the finished samples were lower than the limits of the identification standards for hazardous wastes—identification for extraction toxicity (GB 5085.3-2007).

The vitrification process is an improvement of the solidification process. The TCLP results show that the leaching rate of the solidified and vitrified product is lower than the environmental protection emission standard. Meanwhile, the thermal stability of the product is excellent. This method has the advantages of low raw material cost and simple operation. However, its long-term stability has not been confirmed.

### 3.5. Resource Utilization for SSD and SSPS

#### 3.5.1. Recovery Treatment

(1) Recovery of valuable metals from SSD

The resource utilization of SSD is mainly to recover valuable metals by direct reduction at high temperature. This method can not only realize the utilization of valuable elements in dust, but also solves the environmental pollution caused by the stacking of dust. In China, the rotary kiln process, tunnel kiln process, and Oxycup shaft furnace process are the basic methods for recovering and treating SSD [47,48,49]. In addition, more research has focused on directly adding the pelletized SSD into molten iron pretreatment, electric arc furnace, converter, submerged arc furnace, and other smelting processes, so as to recover valuable metal elements in recent years [50,51,52,53,54]. The main utilization process of SSD in China is shown in Table 9.

The research on the recovery and treatment of SSD in foreign countries has been carried out earlier, and some more advanced recovery processes have been developed. The following Table 10 lists the basic equipment and characteristics of direct reduction recovery for SSD in foreign countries.

(2) Recovery of valuable metals from SSPS

Normally, there are two methods to recover valuable metals from SSPS, namely the hydrometallurgical leaching process and the pyrometallurgical reduction process [60]. Hydrometallurgical leaching is divided into acid leaching and ammonia leaching, respectively. The acid leaching agents mainly include nitric acid, sulfuric acid, and hydrochloric acid. The selection of leaching agent depends on the characteristics of SSPS. Ammonia leaching agents mainly include ammonium carbonate and ammonium hydroxide [61]. Liu et al. [62] used sulfuric acid to leach toxic metals including manganese, nickel, and chromium from SSPS. The results showed that the sludge was disposed of harmlessly and the recovery of iron, manganese, chromium, and nickel was realized. The wastewater produced in the process of recovery can be directly reused after meeting the standard. Zhang et al. [63] used H_2_SO_4_ to leach the heavy metal ions in SSPS. Subsequently, NaHSO_3_ and NaOH solution were added to the leaching mixture to obtain Cr(OH)_3_ and Ni(OH)_2_ products. The recovery rates of Cr and Ni were 93.9% and 94.7%, respectively.

However, hydrometallurgical leaching of valuable metals in SSPS is associated with several drawbacks, including a complex process, large consumption of leaching solution, complicated operation, and difficult separation of iron and chromium when the iron content is high. The hydrometallurgical leaching process, therefore, exhibits significant limitations when considering its application in large-scale industrial treatment.

The pyrometallurgical reduction refers to the process of separating metals from gangue or other impurities, involving a series of physical and chemical reactions of raw materials. For example, the ore or concentrate is treated under a high-temperature condition to recover metals. In recent years, the pyrometallurgy reduction process has been widely used in the field of harmless treatment for SSPS. Zhang et al. [64] proposed a new method for recovering metals from ferritic SSPS using municipal sludge as the reducing agent. The effects of temperature, municipal sludge content, and reaction atmosphere on the recovery performance were studied. The results showed that the recovery rates of Fe, Cr, and Ni reached their highest value of 70.1%, 53.7%, and 60.3%, respectively, under the condition of 650 °C and a 5% blend ratio. Wu et al [65] carried out the harmless treatment and resource utilization of SSPS by direct reduction and magnetic separation. Firstly, Fe^3+^, Cr^3+^, and Ni^2+^ were transformed into Fe, Cr, and Ni by direct reduction at 1473 K. After that, the reduction products were subjected to separate metals and non-metals by magnetic separation. The recovery rates of Fe, Cr, and Ni were 95.3%, 88.7%, and 97.53%, respectively. Wang et al. [66] proposed a novel method to prepare low carbon and low sulfur Fe-Cr-Ni-Si alloy by using high CaSO_4_-containing SSPS. Firstly, the sludge was pre-reduced by the carbon at 1100 °C, and then the pre-reduced sludge was mixed with silicon powder for deep reduction, melting, separation, and desulfurization at 1550 °C. Finally, Fe-Cr-Ni-Si alloy with a carbon content of 0.513 wt.% and a sulfur content of 0.003 wt.% was prepared. The contents of Fe, Cr, and Ni in the alloy were 59.600 wt.%, 15.440 wt.%, and 13.570 wt.%, respectively, and the corresponding recovery rates were 97.99%, 97.61%, and 98.65%, respectively.

The pyrometallurgical reduction process is comparatively simpler than the hydrometallurgical leaching process, while simultaneously achieving a high metal recovery rate. However, the requirement for elevated temperatures in pyrometallurgical reduction leads to increased costs and energy consumption.

#### 3.5.2. Preparation of Value-Added Materials

The SSD and SSPS contain SiO_2_, Al_2_O_3_, CaO, Fe_2_O_3_, CaSO_4_, and other components that bear certain similarities to the raw materials used in the preparation of building materials. The utilization of SSD and SSPS for preparing building materials represents a significant path towards large-scale industrial application of these substances, encompassing the preparation of bricks, ceramsite, ceramic skeletons, glass-ceramics, nucleating agents, and so on.

Zhang et al. [67] used SSD and Cr_2_O_3_ as raw materials to synthesize black pigments by sintering at 1200 °C for 30 min, and then these were added to the ceramic matrix to produce black ceramic tiles. The results showed that the performance of ceramic tiles was best when the sintering temperature, sintering time, and pigment ratio were set as 1200 °C, 30 min, and 8%, respectively. The compressive strength of ceramic tiles exceeded the minimum compressive strength of China’s national standard for the polished tiles, and the toxic substances in the leaching solution also met the national limits. Zeng et al. [68] added reducing agent into SSPS for preparing sintered bricks. The results showed that the Cr^6+^ and other heavy metals in SSPS were solidified in the brick without being leached out, demonstrating the good stability of the prepared bricks. Zhu et al. [69] prepared ceramsite by mixing SSPS and clay at a mass ratio of 20:80 and heating temperature of 1100 °C. The results showed that the average compressive strength of the ceramsite was 810 N, and the leaching rate of Cr and Ni was far lower than the national standard. Cheng [70] mixed SSD and incinerator fly ash in a mass ratio of 1:9 to prepare glass-ceramics. It was found that the main phases of the prepared glass-ceramics were hornfels, gabbro and siderite during the temperature range of 800~1100 °C. After 5 h of heat treatment, the glass-ceramics exhibited exceptional performance with a compressive strength of 1.2 GPa, a density of 3.2 g/cm^3^ and an impressively low porosity of merely 0.6% at 900 °C. Consequently, the prepared glass-ceramics hold great potential for utilization as construction components or refractory materials. Yang et al [71] studied the crystallization behavior and properties of glass-ceramics in a CaO-MgO-SiO_2_-Al_2_O_3_ system using SSPS as the nucleating agent. The experimental results showed that the crystallization temperature of glass-ceramics was decreased by 110.8 °C, the grain size was refined to 2 μm, the Vickers hardness was increased from 4890 MPa to 7580 MPa, and the water absorption was reduced from 1.21 ± 0.10 wt.% to 0.04 ± 0.01 wt.% by adding 14 wt.% SSPS as the nucleating agent. Therefore, the SSPS can be used as a nucleating agent to improve the crystallization and mechanical properties of glass-ceramics.

### 3.6. Development Trends of Harmless Treatment and Resource Utilization for SSD and SSPS

The comprehensive utilization of stainless steel dust (SSD) and stainless steel pickling sludge (SSPS) mainly includes harmless treatment and resource utilization. Typically, the harmlessly treated SSD and SSPS are usually directly disposed of in landfill, which poses environmental risks. The toxic elements can infiltrate water systems during landfill, leading to contamination of both water and soil. Moreover, direct landfill has caused the waste of secondary resources, particularly iron, nickel, chromium, and other valuable metals in SSD and SSPS, which belongs to the initial stage of comprehensive utilization for SSD and SSPS. Therefore, resource utilization in the future should be the key research and development direction for the comprehensive utilization of SSD and SSPS.

The resource utilization of SSD and SSPS includes the recovery of valuable metals and the preparation of value-added materials. It is concluded that the recovery of valuable metals in SSD and SSPS mainly includes two processes, namely hydrometallurgical leaching and pyrometallurgical reduction. The hydrometallurgical leaching process is characterized by its complexity, high cost, extensive equipment requirements, and significant consumption of leaching agents. Consequently, it may not be suitable for large-scale industrial production. The pyrometallurgical reduction process is currently widely employed. Among various pyrometallurgical reduction processes, the method of direct return to production has garnered significant research attention in recent years. It directly uses the high-temperature environment of industrial kilns to recover valuable metals from SSD and SSPS. Apart from the kiln itself, this method exhibits minimal carbon emissions, thereby avoiding secondary pollution. Additionally, this method offers the advantages of low investment and rapid implementation. Therefore, research into resource utilization for SSD and SSPS should focus on the method of direct return to production in the future. At the same time, the efficient utilization of calcium oxide, magnesium oxide, and other components in SSD and SSPS should be strengthened. In addition, attention should also be paid to energy conservation and emission reduction during the production process; that is, we should not only achieve the resource utilization of valuable metal resources and components, but also prevent secondary pollution to the environment.

## 4. State of the Art—Typical Nonferrous Metallurgical Hazardous Waste

Aluminum is the most widely used metal material with the highest yield in nonferrous metallurgy. Aluminum ash (AA) is the main industrial solid waste produced in the process of electrolytic alumina, metal aluminum processing, and waste aluminum recycling. With the continuous increase in aluminum production, the stock of AA is also increasing. Therefore, the harmless treatment and resource utilization of AA are urgent topics to investigate.

### 4.1. Formation of AA

According to the different production processes and content of metal aluminum in AA, it is usually divided into primary aluminum ash (PAA) and secondary aluminum dross (SAD) [72]. PAA is a non-melted scum formed on the surface of the aluminum liquid during the electrolysis process, resulting from its exposure to air. It exhibits a white color; thus, it is commonly referred to as white ash. SAD refers to the residual dross of PAA and waste aluminum after recycling aluminum. It appears black in color, and it is also known as black ash [73].

### 4.2. Chemical Composition and Hazards of AA

(1) Chemical composition of AA

The chemical compositions of PAA and SAD have obvious differences. Table 11 shows the main chemical compositions of PAA and SAD [74,75]. It can be seen from the table that PAA contains a higher content of aluminum metal, ranging from 30% to 70%. On the contrary, the content of metal aluminum in the secondary aluminum ash is low, and the aluminum contained in it mainly exists in the form of alumina. Hence, metal aluminum mainly exists in PAA, so it is normally used for the recovery of metal aluminum. SAD is mainly utilized for the preparation of activated alumina and value-added materials, including building materials, refractory materials, and flocculants. Therefore, both PAA and SAD possess significant value for resource utilization [76].

(2) Hazards of AA

The hazards of AA are mainly derived from two of its ingredients: (1) fluoride- and chloride-based salts: these were added as refining flux during the PAA smelting recovery process and are mainly used to minimize the oxidation of aluminum and to facilitate the agglomeration and separation of metal. But these salts usually have typically high leachability and easily cause the penetration of toxic ions into the soil and groundwater, resulting in serious environmental problems [77]. (2) Aluminum nitride (AlN): related research shows that the formation of AlN in SAD is caused by the reaction between molten aluminum and atmospheric nitrogen. Similarly, it also has a high chemical activity, and it generates harmful and poisonous ammonia gas when it comes in contact with water or even humid air, which seriously affects the ecological environment and people’s health [78].

Based on the obvious toxicity and reactivity, AA (including PAA and SAD) was officially included in the National Hazardous Waste List (2021 Edition) [79]. Because it was included in the hazardous waste list, it cannot be stored for a long time or directly landfilled. Hence, it is necessary to carry out some feasible and suitable for industrialization harmless measures and resource utilization technologies for AA to meet the requirement of environmental protection.

### 4.3. Toxicity Extraction Test of AA

According to the identification standards for hazardous wastes—identification for extraction toxicity (GB 5085.3-2007), Dai et al. [80] detected the inorganic fluoride leaching toxicity of SAD samples from ten enterprises in the Yangtze River Delta region (mainly distributed in Zhejiang and Jiangsu Province, China). The results are shown in Table 12.

The leaching concentration limit of inorganic fluoride is 100 mg/L according to GB 5085.3-2007. It can be seen from the table that all SAD samples had fluoride leaching, and the leaching mass concentration of fluoride (calculated as AlF_3_) ranged from 181 to 1910 mg/L, which exceeds the concentration limit specified in the standard. Among the 10 samples, the fluoride leaching mass concentration of 8 samples exceeded 500 mg/L, while the fluoride leaching mass concentration of the remaining 2 samples even exceeded 1500 mg/L. The fluoride leaching mass concentration of sample No. 9 was the highest, which was 19.1 times of the standard concentration limit. Even the lowest leaching mass concentration of sample No. 6 was 1.81 times of the standard concentration limit. In general, the inorganic fluoride leaching concentration of SAD sample seriously exceeded the standard, and the sample behaved as an inorganic fluoride, leaching toxicity. Hence, SAD was classified as a hazardous waste.

### 4.4. Harmless Treatment of AA

Currently, the alumina plant directly recycles PAA, while SAD is discharged and managed in accordance with hazardous waste standards. Consequently, the harmless treatment of AA mainly focuses on SAD. The harmful components of SAD are aluminum nitride, chloride, and fluoride salts [81]. Aluminum nitride is derived from the reaction of aluminum with nitrogen in air under high-temperature melting conditions. Fluorine and chlorine salts are mainly derived from salt flux, for example, NaCl-KCl, CaF_2_ and NaF [82].

#### 4.4.1. Removal of Aluminum Nitride

Aluminum nitride can not only react with water to form aluminum hydroxide and ammonia, but also decompose into alumina and nitrogen at high temperature. Therefore, there are two main processes to remove aluminum nitride, namely the high-temperature roasting method and hydrolysis method.

(1) High-temperature roasting method

**Figure 1:**
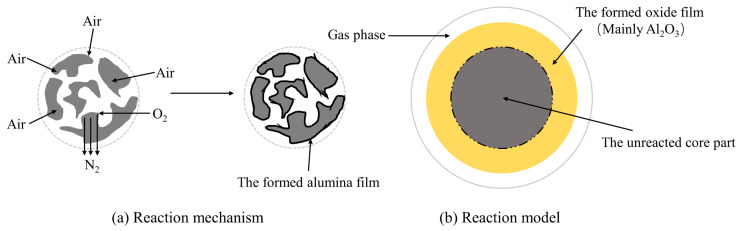
(**a**) Reaction mechanism diagram of O_2_ and AlN in high-temperature roasting; (**b**) model diagram of roasting O_2_ and AlN [85].

The mechanism of high-temperature roasting denitrification is shown in Figure 1a. The surface layer of AlN particles first reacts with O_2_ to form the AlN-Al_2_O_3_ core–shell structure, and then O_2_ diffuses to the AlN-Al_2_O_3_ interface through the Al_2_O_3_ layer and oxidizes AlN, so that the Al_2_O_3_ layer gradually thickens. Due to the large difference in thermal expansion coefficient between Al_2_O_3_ and AlN, the core–shell structure gradually breaks with the increase in temperature and then results in the exposure and reoxidation of AlN [83,84]. As shown in Figure 1b, this process is a gas–solid reaction model with alternating external and internal diffusion.

Wang et al. [86] used a cryolite pyrometallurgy process to remove AlN in SAD. The experiment proved that cryolite can effectively promote the oxidation of AlN, and the removal rate of AlN was as high as 94.71% under optimized conditions. Tang [87] used a muffle furnace to remove AlN in SAD. The results showed that the denitrification rate of SAD was only 22.18% at 700 °C. The denitrification rate was increased up to 90.24% by adding sodium carbonate and sodium peroxide to destroy the product layer. It can be seen that the addition of oxidant can effectively improve the removal rate of AlN. Li et al. [88] investigated the effect of calcination process on the removal of AlN from SAD. The results showed that the removal rate of AlN was 93.8% under the optimized conditions.

The high-temperature roasting method consumes a substantial amount of energy, and it presents challenges, such as incomplete oxidation and indistinct composition of gas products. Additionally, the emission of nitrogen oxides resulting from this process contributes to negatively affect the environment. Currently, there is a paucity of studies investigating the gas products generated through the high-temperature roasting method, posing challenges for its large-scale industrial application.

(2) Hydrolysis method

**Figure 2:**
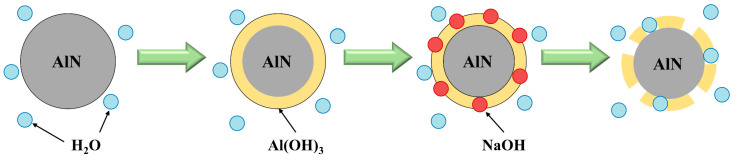
AlN hydrolysis method with the addition of NaOH in SAD [90].

Industrial water is the most widely used solvent for the hydrolysis method. The AlN reacts with the industrial water to produce ammonia, which has high solubility in water to achieve the denitrification of SAD. The hydrolysis of AlN is mainly affected by temperature, time, and solid–liquid ratio. Li et al. [89] found that the hydrolysis product of AlN would wrap itself, thereby inhibiting the hydrolysis reaction. Lv et al. [90] studied the hydrolysis behavior of AlN in SAD and found that the reaction firstly formed hydrated alumina on the surface of AlN. Subsequently, the formed hydrated alumina was converted into Al(OH)_3_, resulting in a slower reaction rate. It was found that the hydrolysis of AlN was significantly affected by the temperature and additive type. The addition of NaOH can effectively enhance the hydrolysis rate by inducing an increase in temperature and creating an alkaline environment. The removal efficiency of AlN reached 96.24% under the following optimum conditions: leaching time of 180 min, leaching temperature of 95 °C, addition of 4 wt% sodium hydroxide, and a liquid-to-solid ratio of 6 mL/g. Figure 2 illustrates the AlN hydrolysis process with the addition of NaOH in SAD. At the beginning of the reaction, the contact conditions of AlN with water are sufficient. As the hydrolysis reaction progresses, the surface of AlN is gradually covered by an insoluble solid product, which then hinders the hydrolysis process. Subsequently, the added NaOH reacts with the protective layer of Al(OH)_3_ to produce soluble NaAlO_2_, leading to the exposure of the AlN surface and the facilitation of the hydrolysis reaction.

The hydrolysis method is a widely employed treatment for the denitrification of SAD due to its advantages of simplicity, low energy consumption, and high removal efficiency. However, it should be noted that the production of H_2_, CH_4_, H_2_S, and other gases cannot be completely avoided during the process. If these gases accumulate around the plant, they may pose significant safety hazards.

There are many inhibitions in the high-temperature roasting and hydrolysis method. The alumina film generated in the process of the roasting method will cover the AlN surface, preventing its further oxidation. For the hydrolysis method, it is also necessary to add additives or adjust the pH value to destroy the Al(OH)_3_ film coating on the surface of AlN, thereby increasing the denitrification rate. At present, the research on the removal of AlN is only to optimize the process conditions. The in-depth study of the AlN removal mechanism and the exploration of more efficient and lower-cost AlN removal process should be the direction for further research.

#### 4.4.2. Removal of Fluoride and Chloride Salts

At present, the common method of removing fluoride and chloride salts in SAD is the washing–precipitation process, which utilizes the different solubilities between salts and other components in SAD.

Abd Aziz et al. [91] used a ball mill to reduce the granularity of SAD and then washed the dross with a solid liquid ratio of 1:4. The removal of chloride salts was completed after 8 days at an ambient temperature. The research conducted by Bao et al. [92] demonstrated that the maximum leaching rates of fluorine and chlorine were 87.38% and 99.02%, respectively under the following optimal process parameters: leaching time of 8 h, leaching temperature of 60 °C, leaching liquid pH of 4, and a liquid–solid mass ratio of 6. Zhang [93] employed the alkaline roasting method to remove fluorine and chloride in SAD, thereby investigating the impact of individual factors on the leaching rate of these elements. Consequently, the leaching rates of fluorine and chlorine reached 94.3% and 98.8%, respectively. Xie et al. [94] carried out an experiment on the high-temperature denitrification and dichlorination of SAD. It was found that the nitrogen and chlorine removal rates reached their highest value of 98.16% and 99.33%, respectively, under the following conditions: calcination temperature of 1300 °C and calcination time of 1 h.

### 4.5. Research Status of Recovery Treatment and Resource Utilization of AA

PAA contains a high content of aluminum, so its recycling is mainly aimed at the recovery of metal aluminum. Furthermore, the recovery treatment of SAD is mainly to prepare activated alumina.

#### 4.5.1. Recovery Treatment of PAA

PAA is a white powder with a high aluminum content ranging from 30% to 70%. Currently, the recovery treatment of PAA mainly involves extracting the high-value metal aluminum [95] by means of electrostatic separation, mechanical screening, and pyrometallurgy. The electrostatic separation method involves recovering PAA by using the difference in conductivity between aluminum and salt. The mechanical screening method involves sieving the ground PAA to separate the aluminum particles from the ash. However, the recovery rate of the previous two methods is relatively low, which is why they are only suitable for recycling cold aluminum ash. The pyrometallurgical recovery utilizes the residual heat from PAA or an external heat source to heat the ash for aluminum recovery, which is widely adopted at present. Depending on whether salt flux is added, the pyrometallurgical recovery methods can be classified into salt-adding processes and salt-free processes [96].

(1) Salt-adding processes

**Figure 3:**
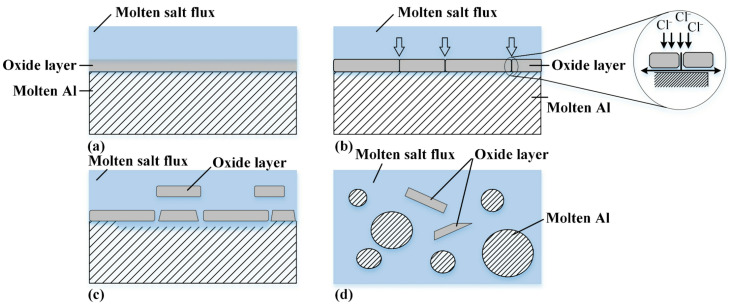
Schematic diagram of oxide layer separation by salt flux [98]: (**a**) Contact of molten salt with oxide layer; (**b**) corrosion of oxide at boundaries and chlorine penetration to oxide/aluminum interface; (**c**) oxide separation in molten salt; (**d**) aluminum droplets.

Salt-adding recovery refers to the addition of salt flux to the furnace when recovering aluminum from PAA. The salt flux corrodes the alumina film on the surface of metal aluminum to achieve the effective recovery of aluminum [97]. Figure 3 depicts a schema of the process of oxide removal from molten aluminum surface by salt flux [98]. As seen in Figure 3a, the molten salt flux is initially in contact with the oxide layer formed on the surface of the liquid aluminum. Then, the chlorine anions in the flux composition attack the oxide layer (Figure 3b). A continuous attack breaks down the boundaries of the oxide grains and accumulates near or at the interface of the aluminum oxide (Figure 3c). Finally, as seen in Figure 3d, aluminum droplets are collected.

The salt-adding process comprises the fried ash method, rotary salt furnace (RSF) method, metal recycling machine (MRM) method, and modified MRM method. The characteristics of different salt-adding processes are shown in Table 13.

Although the salt-adding process is still the main method of PAA pyrometallurgical recovery, the social benefits of the salt-adding process are significantly reduced due to its high energy consumption of the equipment and the secondary pollution of salts. With the increasingly tight energy supply and environmental problems, the research and development of environmentally friendly salt flux should be strengthened.

(2) Salt-free processes

The fundamental principle of the salt-free process is to destroy the oxide film on the surface of aluminum at elevated temperature (700~950 °C) without adding salt flux. The press process is a traditional salt-free process, during which the molten aluminum can be squeezed out under a certain pressure by a slag extrusion machine. However, the recovery rate of this process is only around 60%. Consequently, alternative salt-free processes, such as the Alcan process, ALUREC process, DROSCAR process, ECOCENT process, and DROSRITE process have emerged in recent years. Table 14 presents the characteristics of various salt-free processes for PAA.

In comparison to the salt-adding process, the salt-free processes offer the advantages of reduced environmental pollution and lower energy consumption. Therefore, they should be considered as a promising direction for future research and development.

#### 4.5.2. Recovery Treatment of SAD

SAD refers to the black–gray granular solid residue that remains after aluminum is recovered from PAA and the dust generated during the production of casting aluminum, commonly known as black ash [110]. The recovery treatment of SAD primarily involves the preparation of activated alumina, which mainly adopts the hydrometallurgical leaching process. It can be categorized into the acid leaching process and the alkali leaching process.

(1) Acid leaching process

The acid leaching process can be roughly divided into three steps: the dissolution of SAD, the precipitation of the filtrate, and the calcination of the precipitate [98]. The commonly employed leaching solutions are HCl and H_2_SO_4_, with the latter often necessitating pretreatment of SAD. However, due to its high corrosiveness, the pretreatment step can typically be omitted when using HCl.

Sarker et al. [111] used an acid dissolution process to recover the alumina from SAD. The effects of various parameters, e.g., temperature, acid concentration, and leaching time on the extraction of alumina were studied. It was found that the purity of the alumina extracted in the study was found to be 99.0% under the following optimum conditions: 4 mol/L of HCl, 120 min of leaching time, and a temperature of 100 °C. Yang et al. [112] studied the leaching kinetics mechanism of SAD in HCl and found that the metal aluminum in SAD could completely react with HCl within 50 s, and that the reaction rate of AlN, Al_2_O_3_, and HCl increased with the increase in temperature. According to the research of Dash et al. [113], it had been observed that the presence of salts, like KCl and NaCl, in SAD significantly influenced the recovering process. However, subsequent removal of these salts facilitated the recovery of approximately 85% alumina with a H_2_SO_4_ mass fraction of only 15%. Mahinroosta et al. [114] presented the synthesis and characterization of high crystalline activated alumina nanopowder from SAD using a novel five-step leaching-based process. Under the optimized leaching conditions, i.e., particle sizes ranging from 38 to 75 mm, a leaching time of 120 min, a leaching temperature of 85 °C, an acid concentration of 5 mol/L, and a liquid-to-solid ratio of 20 mL/g, the extraction efficiency of alumina reached approximately 83%.

(2) Alkali leaching process

The basic process of alkali leaching is similar to that of acid leaching, but the reaction between the alkali leaching agent and SAD is relatively complex. In the process of alkali leaching, AlO_2_^−^, Al_2_O_3_, Al(OH)_3_, and other compounds are generated due to the different types and amounts of leaching agents [115]. Tripathy et al. [116] used the soda-roast and dilute alkali leaching route to recover alumina from SAD. The recovery rate of alumina could reach 90% when SAD with particle size of less than 150 μm was roasted at 800 °C and leached in the sodium hydroxide solution for 1 h. The alumina extraction from SAD was performed using the alkali leaching process by Li et al [117]. The experimental results demonstrated a remarkable 98.6% dissolution rate of alumina under the following conditions: a solid–liquid mass volume ratio of 0.08 g/mL, an alkali mass concentration of 248 g/L, a reaction temperature of 250 °C, a reaction time of 3 h, and a reaction stirring speed of 400 r/min. 

For the waste liquid produced in the acid leaching and alkali leaching process, the salt can be recovered by evaporation, concentration, and crystallization after effective neutralization. The condensed water can be returned to the leaching process for reuse. The acid leaching process is characterized by its simplicity, low cost, and minimal discharge of waste residue. However, the purity of the aluminum oxide is relatively low which can be attributed to the dissolution of various metal oxides (such as magnesium oxide, calcium oxide, and zinc oxide) into the leaching solution. Consequently, it results in a complex composition of the leaching solution and poses challenges for its treatment. On the other hand, the alkali leaching process yields a comparatively higher purity of the product; however, it requires a significant amount of alkali consumption, which leads to increased costs.

#### 4.5.3. Preparation of Value-Added Materials from SAD

SAD mainly contains Al_2_O_3_, iron, silicon, magnesium oxide, potassium, sodium calcium, magnesium chloride, etc., which can be used as renewable resources for comprehensive utilization. Currently, the application of SAD in the synthesis of value-added materials is primarily focused on building materials, refractory materials, and flocculants.

(1) Preparation of building materials

The alumina, magnesium oxide, and other compounds in SAD have the characteristics of high temperature resistance, hardness, and compression resistance, and can be used as one of the raw materials for the preparation of building materials. Foo et al. [118] tried to completely use industrial waste to prepare mullite ceramics. The fly ash and SAD were mixed at a Al_2_O_3_:SiO_2_ molar ratio of 3:2, and then they were compacted and sintered at 1500 °C to obtain mullite ceramics with excellent thermal expansion properties. Ewais et al. [119] utilized SAD, aluminum mud, and pure Al_2_O_3_ for the synthesis of calcium aluminate cement, and the incorporation rate of SAD reached an impressive 45.53% in the process. Mailar et al. [120] used SAD as the raw material in the preparation of concrete and found that the mechanical strength and durability were enhanced by incorporating 20% mass fraction of SAD, rendering the concrete suitable for high-temperature pouring conditions. Liu et al. [121] successfully prepared porous ceramics with hierarchical pores and high strength by using SAD and quicklime as the main raw materials. It exhibited a compressive strength ranging from 12.4 to 51.0 MPa, while maintaining a porosity level between 63.7% and 79.6%. Furthermore, through an adsorption experiment utilizing malachite green solution, the potential application of these prepared porous ceramics as effective adsorbents was confirmed. Li et al. [122] proposed a novel, simple, and versatile technique to prepare hierarchically porous ceramics using SAD as the sole raw material, namely the hydrolysis-induced simultaneous foaming and coagulation casting method. Low sintering shrinkage of 1.38–3.60% for the prepared ceramics allows this highly effective and low-cost method to be easily promoted for industrial production.

(2) Preparation of refractory materials

SAD contains a large amount of alumina with a high melting point, which is an ideal raw material for the production of refractory materials. Therefore, the use of harmlessly treated SAD for the production of refractory materials can not only help to realize the recovery of secondary resources, but also reduces the production cost of refractory materials. Li et al. [123] successfully prepared high-alumina refractory materials by sintering SAD with the main crystalline phase of MgAl_2_O_4_ and small amounts of CaAl_2_O_4_ at 1530 °C. Adeosun et al. [124] used SAD and kaolin as raw materials to prepare refractory bricks, investigating the influence of SAD content on their performance. The results revealed that the prepared refractory bricks exhibited exceptional properties within a mass fraction ranging from 40% to 70% for SAD, achieving a remarkable refractoriness of up to 1200 °C. Liu [125] et al. prepared lightweight refractory materials with pretreated SAD and fly ash as the main raw materials. The prepared samples exhibited a linear shrinkage after firing of 1.82%, an apparent porosity of 60.57%, a bulk density of 0.95 g/cm^3^, and a compressive strength of 4.18 MPa. According to the research of Yu [126], magnesium aluminate spinel refractories were prepared by adding magnesia into SAD through roasting. The effects of magnesia content, sintering temperature, and holding time on the properties were investigated. Under the optimal conditions, the magnesium aluminate spinel refractories exhibited an average flexural strength of 106.72 MPa and achieved a maximum bulk density of 3.42 g/cm^3^.

The utilization of SAD for the production of refractory materials significantly reduces the manufacturing cost compared to conventional methods. Moreover, a substantial dosage of SAD is employed in this process, thereby effectively minimizing the stockpiling amount. However, SAD contains a higher content of impure components, which can easily lead to the formation of a glass phase or eutectic point materials. Consequently, its high-temperature properties are impacted. Additionally, the pretreatment process technology route is characterized by complexity and a limited industrial application level, necessitating further urgent investigation.

(3) Preparation of flocculants

The basic aluminum chloride (BAC), polyaluminum sulfate (PAS), and polyaluminum chloride (PAC) are widely used as high-efficiency flocculants [127]. A flocculant has a strong coagulation ability and powerful water purification ability. It demonstrates excellent application effects for removing impurities, sterilizing, and eliminating odors from water. SAD contains a large number of aluminum and silicon elements that can form charged micelles in water to accumulate suspended particles through the action of electrostatic force. Therefore, it can be considered cost-effective to use SAD for preparing high-efficiency flocculants.

Shi et al. [128] used SAD as a raw material to prepare PAC by a series of processes including water washing pretreatment, hydrochloric acid leaching, and polymerization maturing. The results demonstrated that the alumina contents and basicity of the prepared PAC were determined to be 9.09% and 46.30%, respectively, meeting the national standards (GB/T 22627-2014) [129]. The PAC products were successfully prepared by Du et al. [130] through acid leaching polymerization using SAD as the raw material. The optimal conditions for preparation included a temperature of 85 °C, a time of 2 h, and a pH value of 3.0. The resulting product exhibited a basicity of 40.36%, an alumina content of 9.74%, and a density of 1.204 g/cm^3^, all of which meets the national standard (GB/T 22627-2014) [129]. Dong et al. [131] employed SAD as a raw material to synthesize a PAC water purification agent and high aluminum composite materials through a three-step process involving hydrolysis, acidolysis, and polymerization. This approach demonstrated the benefits of simplicity and low production cost, while effectively transforming waste into valuable resources.

The successful preparation of building materials, refractory materials, and flocculants not only provides a low cost and highly competitive product, but also facilitates the utilization of waste resources, thereby providing an effective approach to enhance the economic benefits of aluminum plants.

### 4.6. Development Trends of Harmless Treatment and Resource Utilization for AA

Aluminum ash (AA) can be broadly categorized into primary aluminum ash (PAA) and secondary aluminum dross (SAD). The resource utilization of PAA primarily focuses on recovering metal aluminum. Pyrometallurgy is a commonly employed method, including salt-adding processes and salt-free processes. Among them, the salt-free processes should be prioritized due to their reduced slag production and metal loss, thereby warranting further development.

The application of SAD is limited by the presence of aluminum nitride and soluble salts (fluoride and chloride salts). The removal of aluminum nitride can be achieved through hydrolysis or roasting methods, while soluble salts can be eliminated using the washing–precipitation process. Currently, the majority of harmless treatments for SAD are focused on optimizing reaction conditions with a lack of comprehensive investigation into the underlying reaction mechanisms. This aspect also represents an important avenue for future research. The aluminum content in SAD is relatively low, and its recovery treatment primarily considers the preparation of activated alumina. The hydrometallurgical leaching process is commonly employed, which can be categorized into acid leaching and alkali leaching. The alkali leaching process exhibits high product purity, albeit at a substantial investment cost and elevated energy consumption. The acid leaching process is characterized by its simplicity and cost-effectiveness; however, it exhibits a relatively low level of product purity. Therefore, the development of an efficient, cost-effective, environmentally friendly, and low-emission recovery process for SAD remains imperative in the future. It is worth mentioning that the potential for using water as a leaching agent has been underexplored. The recent literature suggests that water leaching could offer a more sustainable and less corrosive alternative, thereby offering an opportunity for a breakthrough in SAD treatment [132,133]. This approach could potentially lower investment costs and energy consumption while maintaining high product purity. The utilization of SAD to prepare value-added materials mainly focuses on building materials, refractory materials, and flocculants. However, the doping amount of SAD utilized in the production is relatively low, necessitating the addition of pure reagents or supplementary materials. Consequently, this leads to increased costs and diminished added value with the products. Therefore, it is imperative to integrate SAD with various types of solid wastes as a substitute for pure reagents to reduce the production costs.

## 5. General Steps for the Comprehensive Utilization of Metallurgical Hazardous Wastes

Based on a comprehensive review of the current research on the harmless treatment and resource utilization of typical ferrous metallurgical hazardous waste and nonferrous metallurgical hazardous waste, this study proposes a systematic approach towards achieving safe disposal and sustainable utilization of metallurgical hazardous wastes.

**Figure 4:**
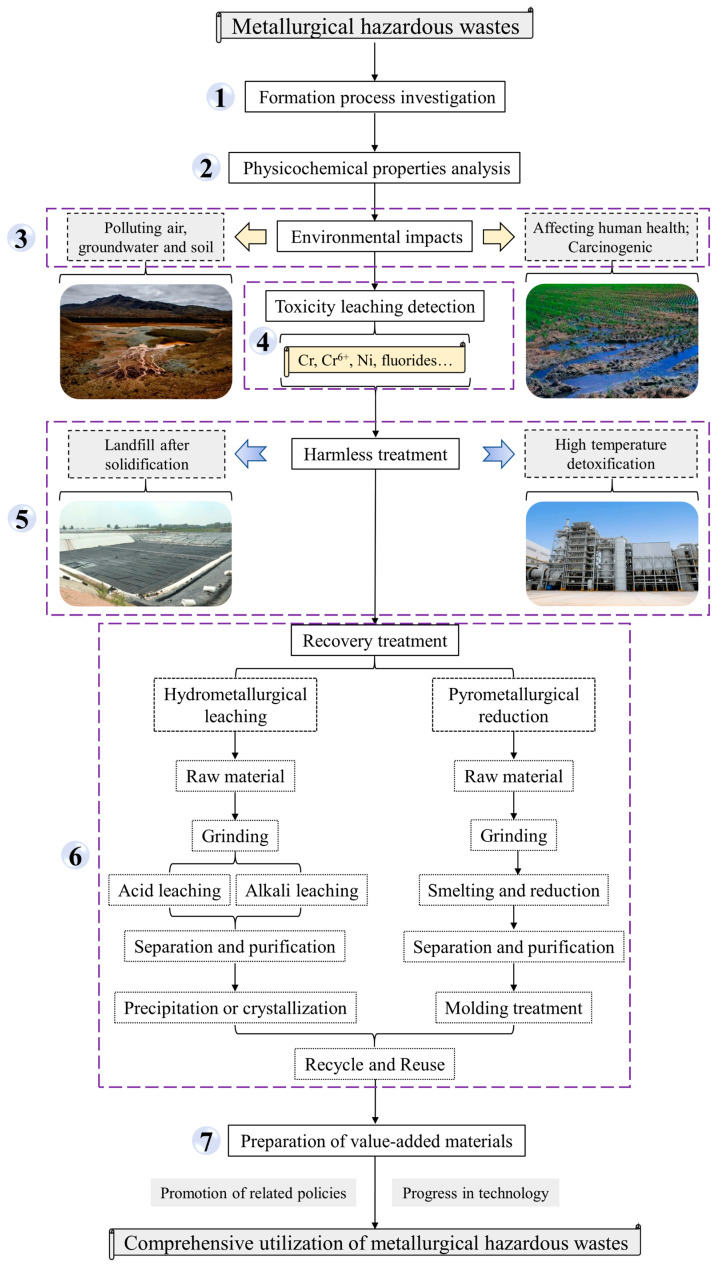
General steps for the comprehensive utilization of metallurgical hazardous wastes.

As shown in Figure 4, the comprehensive utilization of metallurgical hazardous wastes is typically carried out through the following seven steps. To begin with, the generation process of metallurgical hazardous waste should be introduced to assess its potential properties. Secondly, a thorough analysis of the fundamental physicochemical properties of metallurgical hazardous waste needs to be carried out. Following this, the environmental impacts of metallurgical waste should be assessed. Fourthly, it is necessary to conduct an evaluation on leaching toxicity for identifying and characterizing the specific harmful elements. Afterwards, appropriate methods should be employed to dispose of the harmful elements and components. In the sixth step, valuable metals in metallurgical hazardous wastes can potentially be recovered through either hydrometallurgical leaching or pyrometallurgical reduction processes. Furthermore, metallurgical hazardous wastes can be utilized as raw materials for preparing value-added materials, such as building and refractory materials, thereby realizing the comprehensive utilization of metallurgical hazardous wastes.

## 6. Conclusions and Outlook

The metallurgical industry can be broadly categorized into two main branches, namely ferrous metallurgy and nonferrous metallurgy. The present study employs stainless steel dust/sludge and aluminum ash as the representative examples of hazardous wastes for ferrous metallurgy and nonferrous metallurgy, respectively. A comprehensive review is conducted on the current state of harmless treatment and resource utilization, leading to the following conclusion and outlook.

Currently, the comprehensive utilization of metallurgical hazardous wastes encounters numerous challenges. In terms of the recovery treatment, the pyrometallurgical reduction process is characterized by substantial energy consumption, a complex procedure, and significant dust emissions. The hydrometallurgical leaching process generates a lot of waste liquid and residue. Additionally, when using metallurgical hazardous wastes to produce value-added materials, it often necessitates the addition of pure reagents, which limits the incorporation quantity of hazardous wastes and increases costs. In the future, a combined approach of hydrometallurgical and pyrometallurgical processes shows promise for achieving higher metal recovery rates and minimizing environmental impact when extracting valuable metals from hazardous waste. Furthermore, the synergistic treatment of various metallurgical hazardous wastes in the production of value-added materials could increase the incorporation amount of these wastes, aligning with the ‘waste control by waste’ principle. It is believed that advancements in policy and technology are crucial for improving the resource utilization level for metallurgical hazardous wastes, thereby facilitating the genuine realization of sustainable development encompassing resources, environment, and economy.

## Figures and Tables

**Table 1:** Characteristics of toxicity detection methods for the hazardous waste.

Leaching Method	China			European Union		US
	HJ/T 299-2007	HJ/T 300-2007	HJ 557-2010	EN 12457-3		TCLP
Leaching agent	1^#^: m(H_2_SO_4_):m(HNO_3_) = 2:1 solution with pH of 3.2	1^#^: First, 5.7 mL glacial acetic acid is added to 500 mL deionized water, then 64.3 mL of 1 mol/L sodium hydroxide solution is added and diluted to 1 L. The pH value of the solution should be 4.93 ± 0.05	Deionized water	Deionized water		1^#^: First, 5.7 mL glacial acetic acid is added to 500 mL deionized water, then 64.3 mL of 1 mol/L sodium hydroxide solution is added and diluted to 1 L. The pH value of the solution should be 4.93 ± 0.05
	2^#^: Deionized water	2^#^: Ice acetic acid solution with pH of 2.64 ± 0.05				2^#^: Ice acetic acid solution with pH of 2.88 ± 0.05
Applicability of different leaching agent	Leaching agent 1^#^ was used to detect the leaching toxicity of heavy metals and semi-volatile organic compounds in samples Leaching agent 2^#^ was used to determine the leaching toxicity of cyanide and volatile organic compounds in samples	If sample pH < 5.0, use leaching agent 1^#^ If sample pH > 5.0, use leaching agent 2^#^	—	—		If sample pH < 5.0, use leaching agent 1^#^ If sample pH > 5.0, use leaching agent 2^#^
Liquid–solid ratio (L/kg)	10:1	20:1	10:1	2:1	8:1	20:1
Leaching time	18 ± 2 h	18 ± 2 h	8 h	6 ± 0.5	18 ± 0.5	6 h
Vibration mode	Vertical	Vertical	Horizontal	Vertical		Vertical

**Table 2:** Some limit values of GB 5058.3-2007 (mg/L).

Items	Cr	Cr^6+^	Ni	Pb	Zn	Inorganic Fluorides (Excluding CaF_2_)
Concentration limit	15	5	5	5	100	100

**Table 3:** Some metal limit values of 40 CFR 261.24 (mg/L).

Items	Cr	Cd	Hg	As	Pb	Zn
Concentration limit	5.0	1.0	0.2	5.0	5.0	—

**Table 4:** Some metal limit values of 91/689/EEC (mg/kg).

Type of Waste	Cr	Cd	Hg	As	Pb	Zn
Inert waste	0.5	0.04	0.01	0.5	0.5	4
Non-hazardous waste	10	1	0.2	2	10	50
Hazardous waste	70	5	2	25	50	200

**Table 5:** Chemical composition of SSD (wt.%).

Ref.	CaO	SiO_2_	MgO	Al_2_O_3_	TFe	Fe_2_O_3_	FeO	Cr_2_O_3_	NiO	ZnO	Na_2_O	K_2_O
[23]	16.70	4.52	3.34	0.36	—	51.54	—	15.19	3.48	0.12	0.47	0.86
[24]	15.01	4.15	2.87	1.13	—	38.09	18.67	13.20	2.73	0.35	0.22	1.00
[25]	14.40	4.80	1.27	0.52	33.82	—	—	9.38	0.44	4.00	0.72	1.17
[26]	11.40	4.10	2.90	0.88	39.00	—	—	13.70	1.50	0.31	0.87	0.63
[27]	12.90	4.81	5.44	0.40	31.60	—	—	14.60	2.79	4.49	0.60	0.97

**Table 6:** Chemical composition of SSPS (wt.%).

Ref.	CaO	SiO_2_	MgO	Al_2_O_3_	Cr_2_O_3_	NiO	CaSO_4_	CaF_2_	Fe_2_O_3_
[8]	8.74	2.60	0.18	0.61	3.04	1.26	8.92	31.20	25.57
[28]	12.81	1.54	—	0.58	3.93	1.37	11.05	15.56	27.00
[29]	7.30	1.80	0.70	—	11.50	3.00	3.00	47.50	25.80
[30]	2.30	6.90	1.30	2.10	5.30	2.30	—	48.00	26.30
[31]	31.95	8.45	—	—	4.55	1.67	—	38.46	23.19

**Table 7:** Extraction toxicity identification result of SSD (mg/L) [26].

Items	Cr	Cr^6+^
SSD	19.40	18.60
Concentration limit (GB 5085.3-2007)	15	5

**Table 8:** Extraction toxicity identification result of SSPS (mg/L) [21].

Items	Cr	Cu	Zn	Mn	Ni
SSPS	15.2	0.14	—	0.044	0.27
Concentration limit (GB 5085.3-2007)	15	100	100	—	5

**Table 9:** Characteristics of recovery methods for SSD in China.

Method	Feature	Advantage	Disadvantage	Ref.
Rotary kiln process	A certain proportion of stainless steel billet grinding debris, reducing agent, and binder are added to SSD. After stirring evenly, it is pressed into pellets. Then, it is sintered at about 800 °C in a rotary kiln. The sintered ball is used as the raw material for electric furnace smelting. At 1600 °C, the oxides of Cr, Ni, and Fe in the dust are fully reduced by C and Si in the molten steel pool.	Low cost; effectively prolongs the furnace’s life	Slag overflow phenomenon	[47]
Tunnel kiln process	SSD is mixed with iron scale, water, and binder evenly. After drying, coke powder reducing agent is added, and then Ni-Cr sponge iron is formed by reduction at high temperature in the tunnel kiln.	Nickel and iron oxides can be fully reduced	Reduction rate of chromium is relatively low	[48]
Oxycup process	SSD, stainless steel oxide scale, coke powder and binder are mixed evenly to form blocks, and then the blocks are loaded into the Oxycup furnace for high-temperature and high-oxygen enrichment smelting, producing high nickel chromium alloy.	Short flow; efficient; environment-friendly	Chromium recovery rate is unstable	[49]
Direct return to production method	SSD is directly added to the hot metal pretreatment, electric arc furnace, converter, submerged arc furnace, and other smelting processes for recovery treatment after pelletizing.	Short flow; high recovery rate of iron; low slagging agent consumption; low cost	Unstable recovery rate of chromium	[50,51,52,53,54]

**Table 10:** Basic equipment and characteristics of recovery methods for SSD in foreign countries.

Method	Basic Equipment and Feature	Advantage	Disadvantage	Ref.
Inmetco	The core equipment of the process is the annular rotary hearth furnace. Firstly, SSD is mixed with coal and water to make pellets. Then, the carbon-containing pellets are reduced to metal pellets at high temperature in a rotary hearth furnace. Finally, the metal pellets are melted and the chromium oxide is reduced by the residual carbon in the pellets to form metal chromium.	Fast heating rate; high reaction rate; high metal recovery rate	Complicated pretreatment; secondary waste and dust	[55,56]
Fastmet /Fastmelt	The core equipment of the process is also the annular rotary hearth furnace. First, SSD is mixed with coal and binder to make pellets. Then, the carbon-containing pellets are dried and put into a rotary hearth furnace for high-temperature reduction.	Short process; small occupation; short reaction time; no secondary pollution	The recovery rate of chromium is unstable, fluctuating between 70% and 90%; high energy consumption	[57]
STAR	The basic device of the process is a blast shaft furnace equipped with a fluidized bed. SSD is injected through an upper tuyere, and then the molten oxide in SSD is reduced to metallic elements in a high-temperature coke-packed bed. Elements with high vapor pressure, including zinc and lead, are evaporated and extracted from the top of the furnace.	High recovery rate of iron, nickel, and chromium	Complex process; small industrial scale	[58]
Plasmadust	The main device of the process is a shaft furnace with a coke-packed bed and a plasma generator. SSD is fed into the sealed and water-cooled primary chamber through an air-locked system, and then it is struck by the plasma beam to obtain an activated state. Subsequently, the activated SSD is reduced at high temperature for the recovery of valuable metals.	High recovery rate of iron, nickel, and chromium; pollution-free	High energy consumption; large electrode consumption; high noise	[59]

**Table 11:** Main chemical composition of aluminum dross (wt.%).

Types	Al	Al_2_O_3_	AlN	MgO (MgAl_2_O_4_)	Others	Ref.
PAA	30~70	10~40	15~30	1~5	5~15	[74]
SAD	2~5	40~50	15~25	—	8~20	[75]

**Table 12:** Extraction toxicity identification result of secondary aluminum ash (in terms of AlF_3_) (mg/L) [80].

Samples	1	2	3	4	5	6	7	8	9	10
Fluoride content	510	621	515	1580	690	181	800	277	1910	669
Concentration limit (GB 5083.3-2007)	Inorganic fluorides (excluding CaF_2_) ≤ 100 mg/L									

**Table 13:** Characteristics of salt-adding processes.

Technology	Features	Advantage	Disadvantage	Ref.
Fried ash method	The PAA and salt flux are added to the inclined iron pan, and manually stir fried in it through the external heat source or its residual heat. After that, the aluminum melt is collected at the bottom of the iron pot.	Simple operation; low cost	Secondary pollution; poor operating environment	[99]
RSF	The PAA and salt flux in the rotary furnace are heated by oil or natural gas. With the rotation and rolling of the furnace body, PAA is fully mixed with the salt flux. The oxide film of alumina is destroyed by the salt flux, promoting the agglomeration of aluminum liquid and its effective separation from alumina. Furthermore, it prevents the further reoxidation of aluminum liquid, thereby improving the recovery rate of aluminum.	Simple operation; high aluminum recovery rate (about 80%)	Large smoke emission; high cost	[100,101]
MRM method	The heated PAA is added to special equipment with a stirring device, and the salt flux is added for continuous heating, so as to maintain the temperature of the PAA and to realize the complete recovery of liquid aluminum. Finally, the liquid aluminum is collected and deposited at the bottom of the container by mechanical stirring.	Fast processing speed; high aluminum recovery rate	High cost	[102]
Modified MRM method	In the modified MRM method, the whole process of stirring and aluminum recovery is carried out under argon protection. The aluminum burning loss rate is reduced to 4%, and the recovery rate is as high as 91%.	Low aluminum loss rate; high aluminum recovery rate (about 91%)	High cost	[103]

**Table 14:** Characteristics of salt-free processes.

Technology	Features	Recovery Rate	Advantage	Disadvantage	Ref.
Press	The hot aluminum dross is extruded by a slag extrusion machine and the molten aluminum is squeezed out under a certain pressure.	About 60%	Low cost; fewer impurities; simple	Low recovery rate	[104]
Alcan	Air and nitrogen are synchronously introduced into the slit between the two electrodes at the bottom of the rotary furnace. The electrode produces an arc to heat the gas to 700~800 °C and partially ionizes it. PAA is melted in a high-temperature atmosphere, and the rotary furnace rotates at the same time. The oxide film is broken under mechanical stirring, leading to the production of aluminum.	About 90%	High recovery rate; low energy consumption	High equipment failure rate; complex procedure	[105]
ALUREC	The rotary melting furnace is used, and the oxygen-rich combustion is carried out with natural gas as the fuel. The temperature required for melting aluminum is reached in a very short time. After melting, aluminum is enriched at the bottom of the rotary furnace, and non-metallic slag floats on the top of the aluminum melt.	About 70%	Good operating environment; easy to control	High cost; large dust emissions	[106]
DROSCAR	The PAA is heated by a DC arc between two graphite electrodes in a rotary furnace, facilitating the separation of molten aluminum through mechanical stirring. Simultaneously, the argon gas protection effectively prevents the reoxidation of the molten aluminum.	About 75%	High recovery rate; high efficiency	High energy consumption; low product purity	[107]
ECOCENT	The heated PAA is added to the centrifuge, and the relevant parameters, including temperature and centrifugal speed, are adjusted. Under the action of centrifugal force, the metal aluminum and alumina are separated.	About 85%	Easy control; low energy consumption	Narrow applicability	[108]
DROSRITE	The oxygen-contained fuel was blown into the rotary furnace, and the temperature in the furnace was maintained at about 973–1073 K. The metal aluminum was separated and extracted from PAA at high temperature with argon as the protective gas. Subsequently, the residue produced by the separation process was heated in an oxygen atmosphere to recover the residual metal aluminum.	About 90%	High recovery rate	Complex procedure	[109]

## Data Availability

The data presented in this study are available on request from the corresponding author. The data are not publicly available due to technical or time limitations.
